# Clinical Significance of Whole-Body Computed Tomography Scans in Pediatric Out-of-Hospital Cardiac Arrest Patients Without Prehospital Return of Spontaneous Circulation

**DOI:** 10.3390/diseases12100261

**Published:** 2024-10-20

**Authors:** Masanori Ishida, Taro Tanaka, Shinichiro Morichi, Hirotaka Uesugi, Haruka Nakazawa, Shun Watanabe, Motoki Nakai, Gaku Yamanaka, Hiroshi Homma, Kazuhiro Saito

**Affiliations:** 1 Department of Radiology, Tokyo Medical University Hospital, 6-7-1 Nishi-shinjuku, Shinjuku-ku, Tokyo 160-0023, Japan; gossip.auction@gmail.com (T.T.); toidai.nakai@gmail.com (M.N.); saito-k@tokyo-med.ac.jp (K.S.); 2 Department of Pediatrics and Adolescent Medicine, Tokyo Medical University Hospital, 6-7-1 Nishi-shinjuku, Shinjuku-ku, Tokyo 160-0023, Japan; smorichi@tokyo-med.ac.jp (S.M.); nakazawa.haruka.2r@tokyo-med.ac.jp (H.N.); shunnnnnn7@gmail.com (S.W.); gaku@tokyo-med.ac.jp (G.Y.); 3 Department of Emergency and Critical Care Medicine, Tokyo Medical University Hospital, 6-7-1 Nishi-shinjuku, Shinjuku-ku, Tokyo 160-0023, Japan; uesugi@tokyo-med.ac.jp (H.U.); honchu@tokyo-med.ac.jp (H.H.)

**Keywords:** computed tomography, pediatric patient, out-of-hospital cardiac arrest, return of spontaneous circulation

## Abstract

**Background.** Whole-body computed tomography (WBCT) is commonly employed for primary screening in pediatric patients experiencing out-of-hospital cardiac arrest (OHCA) without prehospital return of spontaneous circulation (ROSC). This study aimed to evaluate the cause of OHCA on WBCT and compare WBCT findings between ROSC and non-ROSC groups in non-traumatic pediatric OHCA cases in an emergency department setting. **Methods.** A retrospective analysis was conducted on 27 pediatric patients (mean age: 32.4 months; median age: 10 months) who experienced non-traumatic OHCA without prehospital ROSC and were transported to our tertiary care hospital between January 2013 and December 2023. WBCT scans were performed to investigate the cause of OHCA, with recorded findings in the head, chest, abdomen, and subcutaneous tissues. **Results.** In all cases, the direct causes of OHCA were undetermined, and WBCT identified no fatal findings. Statistical comparisons of CT findings between the ROSC and non-ROSC groups revealed significant differences. The non-ROSC group had a higher incidence of brain swelling, loss of cerebral gray-white matter differentiation, symmetrical lung consolidation/ground-glass opacity, cardiomegaly, hyperdense aortic walls, narrowed aorta, gas in the mediastinum, and hepatomegaly compared to the ROSC group. **Conclusions.** Although WBCT did not reveal the direct cause of OHCA, several CT findings were significantly more frequent in the non-ROSC group, including brain swelling, loss of cerebral gray-white matter differentiation, symmetrical lung consolidation/ground-glass opacity, cardiomegaly, hyperdense aortic wall, narrowed aorta, gas in the mediastinum, and hepatomegaly. These findings, resembling postmortem changes, may aid in clinical decision making regarding the continuation or cessation of resuscitation efforts in pediatric OHCA cases.

## 1. Introduction

Out-of-hospital cardiac arrest (OHCA) in pediatric populations is a rare but critical event with significant implications for clinical outcomes and public health. As of 2024, the Tokyo metropolitan area had a population of approximately 14 million, including about 2 million individuals under the age of 19 [1]. In 2020, the incidence of OHCA in this age group accounted for just 1.3% of all emergency transport cases, reflecting its rarity (160 out of 12,338 cases) [2]. Specifically, the incidence rates were 0.9 per 10,000 for males and 0.65 per 10,000 for females.

Despite its infrequency, pediatric OHCA presents unique challenges in clinical management. In Japan, the causes of pediatric OHCA often remain undetermined, highlighting the need for a more structured approach, such as the implementation of a Child Death Review system to investigate sudden unexpected deaths in children [3]. Accurate identification of the underlying causes is crucial not only for understanding the etiology but also for informing appropriate resuscitation strategies.

To support treatment decision making in time-critical situations, identifying early available and easily applicable predictors of outcome and survival after OHCA is important. This is particularly crucial in OHCA patients due to the post-cardiac arrest syndrome (PCAS) that occurs after OHCA. PCAS is a highly complex and multifaceted condition characterized by inflammation and quality of perfusion (i.e., ischemia and reperfusion), causing organic functional damage that is often irreversible over time [4]. Therefore, timely and accurate decision making is critical in managing these patients.

A recent survey conducted between November 1 and 12 December 2022, across 502 pediatric training facilities and emergency centers in Japan, reported that 96% of these institutions performed computed tomography (CT) scans on pediatric OHCA cases they received. This rate was comparable to that of routine blood tests and blood gas analyses [5]. The use of CT, particularly whole-body CT (WBCT), has been recognized as a valuable tool in identifying abnormal findings that can guide life-saving interventions and the development of treatment plans. However, in cases where resuscitation is unlikely to succeed, CT findings may also provide essential information to support the difficult decision to cease resuscitation efforts—a decision that requires clear communication with the family, who may be in shock and struggling to comprehend the situation.

Recent advancements in CT technology have further expanded the potential applications of CT in clinical settings, particularly in cardiovascular imaging. One notable innovation is photon-counting CT, which represents a significant leap forward in imaging capabilities. These technological developments in non-invasive imaging are reshaping our approach to cardiovascular prevention and diagnosis [6]. Photon-counting CT offers several advantages over conventional CT, including improved spatial resolution, reduced radiation dose, and enhanced material differentiation. These features could potentially improve the detection of subtle abnormalities in pediatric OHCA cases, providing more detailed information about cardiac and vascular structures. While the application of photon-counting CT in emergency settings is still evolving, its potential to enhance diagnostic accuracy in time-critical situations like OHCA is promising and warrants further investigation.

Although CT is widely utilized for screening potential causes of cardiac arrest [7,8,9,10], its specific role in pediatric OHCA cases—especially among those who did not achieve a return of spontaneous circulation (ROSC) before arriving at the emergency department (ED)—has not been fully elucidated. The clinical utility and implications of CT in these scenarios remain underexplored.

This study aims to assess the utility and clinical implications of CT, particularly WBCT, in pediatric OHCA cases where prehospital ROSC was not achieved. By investigating the WBCT findings in these cases, we seek to contribute to a better understanding of how WBCT can inform resuscitation efforts and decisions regarding the termination of resuscitation in the ED setting.

## 2. Materials and Methods

This study was conducted at Tokyo Medical University Hospital, a 904-bed tertiary care academic medical center in central Tokyo. The hospital’s ED handles about 1800 transported patients per year. The ED is equipped with dedicated pediatric resuscitation bays and has 24/7 access to CT imaging. For pediatric OHCA cases, patients undergo WBCT scanning if deemed necessary by the attending physician, with the CT suite located adjacent to the resuscitation area to minimize transfer time.

This retrospective study was conducted in accordance with the principles outlined in the Declaration of Helsinki. Approval was obtained from the institutional review boards (T2023-0192), and the requirement for informed consent was waived.

### 2.1. Subjects

We retrospectively reviewed pediatric patients who experienced non-traumatic OHCA between January 2013 and December 2023. Inclusion criteria were patients transported to the ED of our tertiary care hospital without ROSC prior to arrival. At the time of this study, the legal age of adulthood in Japan was defined as 18 years, per the Civil Code. Consequently, this study focused on individuals under 18 years of age. The determination of whether the cause of OHCA was traumatic or non-traumatic was made by emergency physicians based on physical examination and information obtained from paramedics, family members, or first responders. Specifically, we adhered to the guidelines outlined by previous reports [11,12]. These references define traumatic OHCA as cases where there is clear evidence of external physical injury as the primary cause of cardiac arrest, such as severe blunt force trauma, penetrating injuries, or falls from significant heights. Non-traumatic OHCA was defined as cases where no such external trauma was evident, and the arrest was presumed to be due to medical causes such as cardiac events, respiratory failure, or other internal pathologies. In cases where the cause was unclear, a multidisciplinary team including emergency physicians and radiologists reviewed the case to make a final determination.

### 2.2. CT Scanning

All patients underwent WBCT without contrast enhancement. During or after cardiopulmonary resuscitation (CPR), WBCT scanning was performed in the supine position, covering the area from just above the vertex to the toes. In scans during CPR, the duration of interruption of chest compressions was less than 10 s. Two types of multi-detector CT scanners were used: a 64-row scanner (Aquilion 64, Canon Medical Systems, Tokyo, Japan) until 30 June 2019 and an 80-row scanner (Aquilion Prime SP, Canon Medical Systems, Tokyo, Japan) from 1 July 2019. Helical scans were performed at a tube voltage of 120 kV. On the Aquilion 64, tube current was set at 200 mA for the head and 300 mA for the body, or 300 mA for the head and 400 mA for the body. The axial slice thickness was 4 mm for the head and 2–5 mm for the body. On the Aquilion Prime SP, tube current was regulated by automatic exposure control, with a slice thickness of 3 mm for the head and 2–5 mm for the body. Image reconstruction, when available, was performed at a thickness of 1 mm. On both scanners, the helical pitch was set at 0.625 for the head and 0.828 for the body, with a maximum field of view of 350–420 mm and a 512 × 512 image matrix.

### 2.3. CT Image Evaluation

WBCT images were independently reviewed via Synapse Picture Archiving and Communication System (Fujifilm, Tokyo, Japan) by two senior board-certified diagnostic radiologists with 14 and 18 years of experience, respectively. In interpreting CT images, diagnostic radiologists could access clinical information (including present illness at admission, past history, and family history), laboratory results (hematological and biochemical tests), arterial blood gas analysis, and available previous images. The radiologists specifically evaluated the WBCT images for findings that could explain the cause of OHCA. Additionally, the presence or absence of the following findings was assessed: brain swelling, loss of cerebral gray-white matter differentiation, hyperdense intracranial venous sinuses, symmetrical or asymmetrical lung consolidation/ground-glass opacity, cardiomegaly, pericardial effusion, hyperdense aortic wall, narrowed aorta, gas in the mediastinum (i.e., in the cardiac cavity, aorta, or superior vena cava), hepatomegaly, dilated inferior vena cava, dilated gastrointestinal tract, gas in upper abdominal organs (i.e., in the liver, kidney, pancreas, or spleen), and dorsal subcutaneous fatty edema. These findings are commonly observed on postmortem CT [13,14] and are, therefore, considered observable during the agonal stage in non-ROSC cases. Consensus was reached on the presence or absence of these findings.

### 2.4. Evaluation Process

In this study, we made the decision to exclude emergency consultations from the evaluation of images and patient data. This choice was made to ensure a standardized and unbiased assessment of the WBCT findings and clinical data. Our rationale was as follows. 1. Standardization. By limiting the evaluation to the predetermined set of radiologists and clinical data, we aimed to maintain consistency in the interpretation of findings across all cases. 2. Blinding. Excluding emergency consultations helped to minimize potential bias that could arise from real-time clinical impressions or preliminary diagnoses made during the acute phase of care. 3. Focus on WBCT findings. Our primary aim was to assess the utility of WBCT in identifying causes of OHCA and predictors of ROSC. We wanted to evaluate these imaging findings independently of other clinical decision-making processes. 4. Retrospective nature. As this was a retrospective study, we sought to analyze the data as they were initially recorded, without the influence of subsequent consultations or interpretations.

### 2.5. Study Period Considerations

Throughout the study period (2013–2023), we adhered to the most current Japanese Resuscitation Council Guidelines, which were updated in 2015 and 2020 [15]. There was a transition of equipment from a 64-row CT scanner to an 80-row CT scanner in July 2019, but there were no significant changes in imaging protocols and minimal impact on CT image evaluation.

### 2.6. Statistical Analysis

Statistical analyses were performed using IBM SPSS Statistics version 29.0. The results were considered statistically significant at *p* < 0.05. Patients were categorized into two groups based on whether ROSC was achieved in the ED. Differences between the ROSC and non-ROSC groups were evaluated using the Mann–Whitney U test. Differences in specific CT findings between the groups were assessed using Fisher’s exact test.

## 3. Results

### 3.1. Clinical Information

During the study period, 27 pediatric patients with non-traumatic OHCA who did not achieve prehospital ROSC were referred to the ED of our tertiary care hospital and subsequently underwent WBCT scanning. Of these, 14 were boys and 13 were girls, with a mean age of 32.4 months (median: 10 months; range: 0.4–166 months). The interval from the last sighting to discovery was estimated based on information provided by family members or the individuals who found the patients. The mean interval time was 3 h and 4 min, with a median of 2 h and 25 min (range: 0 to 9 h and 20 min).

Cardiopulmonary resuscitation was performed in all cases, before and after arrival at the hospital. Eight patients achieved ROSC in the ED and were subsequently hospitalized, whereas 19 patients did not achieve ROSC and were pronounced dead in the ED. We analyzed differences between the non-ROSC and ROSC groups in terms of age, sex, presence of past medical history, location of discovery (home or elsewhere), whether the patient was found after bedtime, estimated time from last sighting to discovery, and the presence of bystander CPR (Table 1). There were significant differences in whether the patient was found after bedtime and estimated time from last sighting to discovery between the two groups. The non-ROSC group was significantly larger for the proportion of patients discovered after bedtime and for the estimated time from last sighting to discovery.

### 3.2. Evaluation of WBCT Findings

WBCT scans were performed during CPR in 25 patients and after the termination of CPR in two non-ROSC cases. In all cases, the direct cause of OHCA remained undetermined by WBCT, and no fatal findings were identified by WBCT.

**Figure 1:**
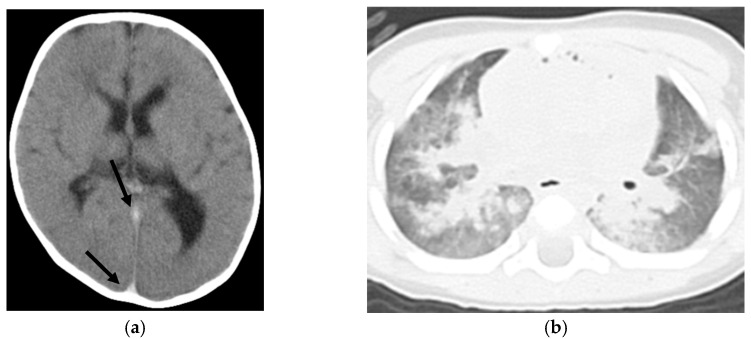

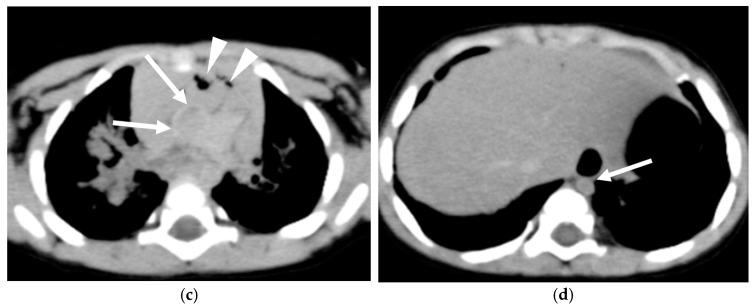
Nine-month-old boy with non-ROSC (case No. 19 in Table S1). (**a**) Observations in the head included brain swelling, loss of cerebral gray-white matter differentiation, and hyperdense intracranial venous sinus (arrows). (**b**) Symmetrical consolidation/ground-glass opacity of the lungs was noted. (**c**) In the mediastinum, findings included cardiomegaly, a hyperdense aortic wall (arrows), and gas in the cardiac cavity (arrowheads). (**d**) A narrowed aorta (arrow) and hepatomegaly were observed.

**Figure 2:**
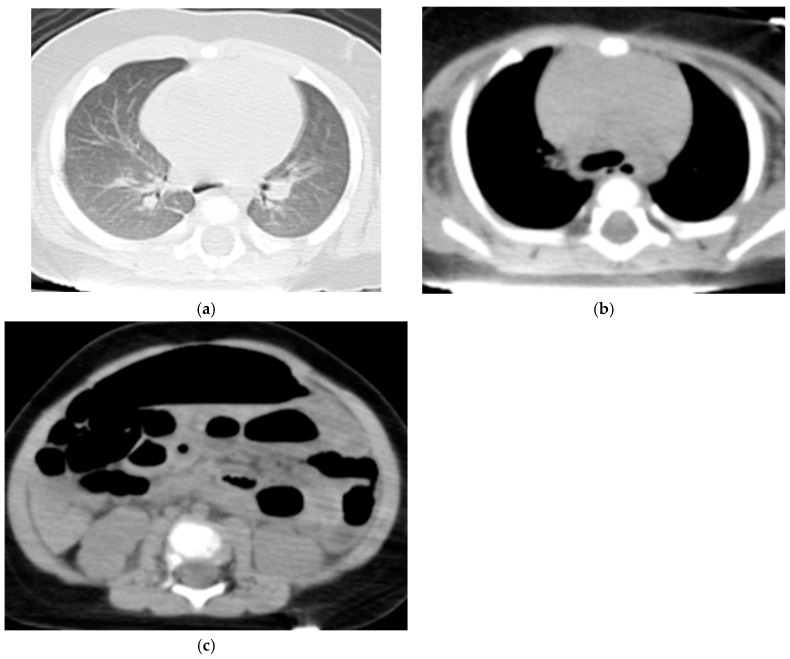
Four-month-old boy with ROSC (case No. 6 in Table S2). (**a**) No symmetrical or asymmetrical lung consolidation/ground-glass opacity of the lungs was observed. (**b**) In the mediastinum, mild cardiomegaly was noted, but no hyperdense aortic wall was identified. (**c**) A dilated gastrointestinal tract was observed.

Supplementary Tables S1 and S2 present clinical information and CT findings of the non-ROSC (Figure 1) and ROSC groups (Figure 2), respectively. The non-ROSC group showed significantly higher incidences of several findings compared to the ROSC group, including brain swelling (84% vs. 12.5%; *p* < 0.001), loss of cerebral gray-white matter differentiation (74% vs. 37.5%; *p* = 0.033), symmetrical consolidation/ground-glass opacity (95% vs. 50%; *p* = 0.017), cardiomegaly (84% vs. 25%; *p* = 0.006), hyperdense aortic wall (84% vs. 0%; *p* < 0.001), narrowed aorta (100% vs. 0%; *p* < 0.001), gas in the mediastinum (80% vs. 0%; *p* < 0.001), and hepatomegaly (79% vs. 12.5%; *p* = 0.002). Neither pericardial effusion nor subcutaneous fatty edema was observed in any case. There were no significant differences between the non-ROSC and ROSC groups in the incidence of loss of cerebral gray-white matter differentiation, hyperdense intracranial venous sinus, symmetrical/asymmetrical consolidation/ground-glass lung opacity, cardiomegaly, hepatomegaly, dilated inferior vena cava, dilated gastrointestinal tract, or gas in the upper abdominal organs. A summary of the differences in the WBCT findings between the non-ROSC and ROSC groups is provided in Table 2.

### 3.3. Hospital Stay Duration

The hospital stay duration differed significantly between the ROSC and non-ROSC groups. All non-ROSC cases (case Nos. 1–19 in Supplementary Table S1) had a hospital stay of 0 days, indicating they died on the day of admission. In contrast, ROSC cases (cases No. 1–8 in Supplementary Table S2) had varying hospital stays: 1, 53, 51, 8, 8, 1377, 34, and 24 days, respectively. For the ROSC group, the mean hospital stay was 194.5 days, with a median of 29 days (range: 1–1377 days). The non-ROSC group uniformly had a 0-day stay. There was a significant difference between the two groups (*p* < 0.001).

## 4. Discussion

This study identifies notable differences in the frequency of certain imaging findings between pediatric OHCA patients who achieved ROSC and those who did not. Specifically, CT features such as brain swelling, loss of cerebral gray-white matter differentiation, symmetrical lung consolidation/ground-glass opacity, cardiomegaly, hyperdense aortic wall, narrowed aorta, gas in the mediastinum, and hepatomegaly were significantly more prevalent in the non-ROSC group. These findings, which resemble postmortem changes, may serve as valuable indicators to guide clinical decision making in challenging resuscitation scenarios. Our investigation into the clinical utility of WBCT during resuscitation in the ED represents a novel approach that may have significant implications for clinical practice, particularly in supporting decisions about continuing or ceasing resuscitation efforts and in facilitating communication with families about prognosis. In our study, we found significant differences in the incidence of specific CT findings between the ROSC and non-ROSC groups. Findings such as brain swelling, loss of cerebral gray-white matter differentiation, hyperdense aortic wall, and narrowed aorta were more frequently observed in the non-ROSC group. These findings may represent physiological alterations during the agonal phase that are similar to normal postmortem changes [13,14,16,17,18]. Following the cessation of circulation, specific changes occur, notably the edematous alterations that elevate the water content in cerebral gray matter, blurring the distinction between gray and white matter. Additionally, there is a narrowing of sulci and ventricles along with brain swelling, predominantly when vasogenic edema is evident. The narrowing of the short arterial diameter and the hyperdense arterial walls likely reflect smooth muscle contraction. These phenomena are analogous to the changes observed as rigor mortis in postmortem examinations [14].

Following circulatory arrest, the vasculature reaches a mean circulatory filling pressure, characterized by a hydrostatic pressure of around 7 mmHg. This pressure exceeds the typical diastolic pressure of the right heart system, prompting a redistribution of blood volume to the right heart system postmortem. Consequently, this can lead to dilatation of the right heart system and the superior vena cava [13,14]. Therefore, clinical signs, such as cardiomegaly and hepatomegaly, indicative of blood shift and pooling in the right heart system, may signal ineffective CPR. These CT findings could inform the potential futility of ongoing resuscitation efforts in specific cases. Additionally, the pressure shift may cause aortic narrowing, whereas symmetrical consolidation or ground-glass opacity in imaging is likely indicative of pulmonary edema resulting from cardiac arrest. It was not clear that inferior vena cava dilation was significantly more frequent in the non-ROSC group in this study, as there were no reports of inferior vena cava dilation as a postmortem change.

Additionally, the primary cause of the findings of gas in the mediastinum (i.e., in the cardiac cavity, aorta, or superior vena cava) or the upper abdominal organs (i.e., in the liver, kidney, pancreas, or spleen) may have been produced by chest compressions [19,20]. Intravascular and intra-organic gas is frequently observed after chest compressions. The causes of this observation include the vaporization of dissolved gasses in the blood, air entry through the infusion route, and the formation of broncho-vascular fistula (pressure trauma to the bronchi and pulmonary vessels) in combination with artificial ventilation [19,21]. The longer the duration of cardiopulmonary resuscitation, the more frequently gas is seen in multiple organs. As reported in postmortem CT, intravenous gas may also relate to intra-osseous needles [22]. Chest compressions can spread intravascular gasses in the arteries in a prograde fashion and the veins in a retrograde fashion. On the other hand, manual ventilation with a bag–valve–mask or similar device supplies air to the body, resulting in extensive dilation of the gastrointestinal tract. This CT finding can be observed on CT owing to manual ventilation [13,14], and there is no significant difference between the ROSC and non-ROSC groups.

On the other hand, no significant difference was found in the intracranial hyperdense venous sinus between the ROSC and non-ROSC groups. Hyperdense venous sinus occurs as a result of circulatory arrest and is known as livor mortis in the postmortem state [23]. In neonates and infants, the brain parenchyma exhibits higher water content and appears with lower attenuation on CT scans. This characteristic facilitates the contrast between brain parenchyma and the developed intradural venous plexus. Consequently, it is plausible that the hyperdense intracranial venous sinuses represent normal physiological variations with minimal correlation to cardiac arrest. Given the challenges of pediatric imaging owing to the lower resolution inherent in pediatric CT modalities [24], radiologists must interpret the CT findings assessed in this study with caution, as their clinical significance may not always be evident.

Although our study did not establish the utility of WBCT in identifying causative factors of OHCA, one of the potential advantages of WBCT is its capacity to detect life-threatening conditions in critical organs such as the heart, brain, or lungs. Nonetheless, in this study, WBCT did not reveal definitive findings that clarified the etiology of cardiac arrest. This aligns with prior postmortem CT research, which indicates that the underlying causes of sudden cardiac arrest in pediatric cases, especially non-traumatic ones, often remain unidentified [25,26,27].

In recent years, postmortem CT has gained popularity in Japan and globally for determining the cause of death as a non-invasive complementary to traditional autopsy, particularly for detecting hemorrhagic, vascular, or other gross pathological lesions. However, its diagnostic accuracy in pediatric cases, especially in non-traumatic deaths, is limited [28]. For example, postmortem CT identified the cause of death in only 12.9% of pediatric cases in which the diagnosis was confirmed by autopsy. In contrast, in 74.1% of cases, postmortem CT did not provide additional diagnostic information, often owing to infectious or cardiovascular causes of death that are not easily identifiable via imaging [27]. Other studies have also reported low diagnostic accuracy for pediatric postmortem CT in cases of natural death, indicating that postmortem CT does not typically provide new insights when the cause of death is unexplained by pathologists [29]. Studies on pediatric postmortem CT have reported that it is often challenging to identify a definite cause of death [27,30,31,32], which is consistent with the present findings. Nevertheless, the absence of significant findings on WBCT can still be clinically valuable, especially in ruling out external causes of cardiac arrest when the circumstances are unclear. Just as postmortem CT can sometimes identify the cause of death, identifying the cause of cardiac arrest on CT could guide immediate and targeted interventions, potentially improving outcomes by preventing delays in treatment in OHCA cases. The role of technology in the management of OHCA cases remains promising and merits further investigation.

A notable concern in pediatric cardiopulmonary arrest cases is the parent’s refusal to discontinue resuscitation procedures. Identification of cardiac arrest is also essential for grief care [33]. In these situations, CT may sometimes be able to explain cardiac arrest and provide important information to help bring some closure to the grieving family. In this perspective, the timing of CT scans in OHCA cases, ideally conducted with family consent, remains a practical challenge. In the previous study, most CT scans were performed during ongoing resuscitation efforts. Previous surveys indicate that 22% of institutions perform CT scans during resuscitation [5], possibly influenced by Japan’s health insurance system, which does not cover postmortem examinations. Consequently, CT scans are often performed during CPR to meet insurance requirements and ensure compensation before death is formally declared.

While our study focused on the utility of WBCT in pediatric OHCA cases, it is crucial to consider the importance of pre-evaluation screening for preventing such critical events. One area of particular interest is the assessment of premature ventricular complexes (PVCs), which are frequently documented in pediatric patients. Historically, PVCs were often associated with an increased risk of sudden death. However, the recent literature suggests a more benign nature of this condition in most cases [34]. While the direct link between PVCs and OHCA risk in children remains a subject of ongoing research, incorporating PVC assessment into preventive strategies could potentially impact the incidence of out-of-hospital cardiac arrest. Future studies should aim to elucidate the clinical relevance and long-term outcomes of frequent PVCs in pediatric populations, particularly in the context of OHCA prevention.

Survival rates for patients experiencing OHCA remain extremely low, underscoring the critical importance of timely intervention. In our study, we observed that the estimated time from the last sighting to discovery was significantly longer in the non-ROSC group compared to the ROSC group. This finding aligns with the growing body of evidence suggesting that the rapidity of initiating and maintaining CPR is crucial in achieving ROSC. Interestingly, recent research has explored the relationship between the volume of interventions by mobile intensive care units (MICUs) and outcomes in OHCA patients. A study reported no significant association between MICU intervention volume and patient outcomes [35]. This suggests that while advanced prehospital care is important, the time to initiation of basic life support measures may be the key determinant in achieving ROSC. These findings lead to a hypothesis that if a considerable amount of time elapses before CPR is initiated, even highly sophisticated prehospital emergency services may not directly translate to improved patient outcomes. However, it is important to note that the relationship between MICU intervention volume and OHCA patient outcomes is complex and multifaceted. Further research is essential to draw definitive conclusions and to fully understand the interplay between time to CPR initiation, advanced prehospital care, and patient survival in pediatric OHCA cases.

Our study spanned nearly 11 years, during which there were changes in resuscitation guidelines, regulations, and imaging equipment. Despite these changes, our primary findings remained consistent throughout the study period. The transition to a more advanced CT scanner in 2019 provided higher-resolution images, potentially enhancing our ability to detect subtle findings in later cases. However, this technological advancement did not significantly alter our main conclusions regarding the utility of WBCT in pediatric OHCA cases.

Hospital stay duration analysis provided additional insights into the outcomes of pediatric OHCA cases. The stark contrast between the ROSC and non-ROSC groups—with the latter uniformly having a 0-day stay and the former showing varied lengths of hospitalization—underscores the critical nature of achieving ROSC in the emergency department. The wide range of hospital stays in the ROSC group (1 to 1377 days) suggests that while achieving ROSC is a crucial first step, it does not guarantee long-term survival or favorable outcomes. This variability in hospital stay duration among ROSC patients likely reflects the heterogeneity of underlying causes, severity of the initial insult, and effectiveness of post-resuscitation care. These findings further emphasize the importance of early and effective interventions in OHCA cases. The ability to achieve ROSC not only prevents immediate mortality but also opens the possibility for extended treatment and potential recovery. However, the wide range of hospital stay durations in the ROSC group also underscores the need for comprehensive post-resuscitation care and long-term follow-up for these patients. Future research should focus on identifying factors that contribute to improved long-term outcomes in pediatric OHCA survivors and developing strategies to optimize post-resuscitation care.

This study presents several limitations. Firstly, this study’s retrospective design and small sample size of 27 cases from a single institution limit the generalizability of our findings. Given the rarity of OHCA without prehospital ROSC, our results should be considered preliminary. This underscores the necessity for larger, multi-institutional prospective studies to validate and extend our observations. Such studies would provide greater statistical power, allow for meaningful subgroup analyses, and more accurately represent the broader pediatric OHCA population. Secondly, the long study period (2013–2023) encompassed changes in guidelines and technology, which may have introduced some variability in our data. Nevertheless, our consistent findings throughout this period suggest that these changes did not substantially impact our main conclusions. We also acknowledge that emergency consultations play a crucial role in real-time clinical decision making. For the purposes of this study, their exclusion allowed us to focus specifically on the predictive value of the WBCT findings in OHCA cases. Future prospective studies may benefit from incorporating these consultations to provide a more comprehensive view of the clinical decision-making process in OHCA cases. Thirdly, the CT findings evaluated were based on known normal postmortem changes; however, other indicators that might predict non-ROSC could exist and were not captured in this study. Thirdly, variability in resuscitation practices, such as epinephrine administration, ventilation methods, and chest compression techniques, might have influenced outcomes, as CPR protocols were not standardized across all cases. Chest compressions were interrupted during WBCT scanning, but the interruption lasted less than 10 s, and we consider that the scanning did not affect resuscitation. Furthermore, the potential influence of other diagnostic measures immediately post-admission, such as echocardiography or abdominal ultrasound, on cardiopulmonary resuscitation procedures was not considered.

The clinical significance of our findings extends beyond mere academic interest. By identifying specific CT features associated with non-ROSC in pediatric OHCA cases, we provide clinicians with additional objective criteria to inform their decision-making process. These imaging biomarkers could potentially be integrated into existing protocols for managing pediatric OHCA, enhancing the objectivity of prognostic assessments. Moreover, these findings may serve as a valuable tool in communicating with families about the severity of the situation, potentially easing the difficult process of discussing cessation of resuscitation efforts when appropriate. As we move forward, it will be crucial to conduct prospective studies to validate these findings and explore how their integration into clinical practice impacts patient outcomes, resource utilization, and family satisfaction with care. The goal is to refine our approach to these challenging cases, balancing the imperative to save lives with the need to avoid futile interventions.

## 5. Conclusions

In conclusion, while WBCT did not reveal the direct cause of OHCA in patients without prehospital ROSC, our evaluation of CT findings during the agonal phase in pediatric OHCA cases identified several findings that were more frequently observed in the non-ROSC group. These include brain swelling, loss of cerebral gray-white matter differentiation, symmetrical lung consolidation/ground-glass opacity, cardiomegaly, hyperdense aortic wall, narrowed aorta, gas in the mediastinum, and hepatomegaly. These imaging features, resembling postmortem changes, may indicate a lower likelihood of achieving ROSC. For parents and families confronted with the sudden cardiac arrest of their child, sharing these CT findings may help communicate the futility of continued resuscitation efforts, potentially easing the process of accepting the situation. Given the critical challenge of managing pediatric OHCA in the ED, WBCT may emerge as a valuable clinical tool in guiding decisions on whether to continue or cease resuscitative efforts in children who have experienced cardiac arrest. Future research should focus on validating these findings in larger, prospective studies and exploring how they can be integrated into clinical decision-making protocols for pediatric OHCA cases.

## Figures and Tables

**Table 1:** Patient characteristics.

	non-ROSC (*n* = 19)	ROSC (*n* = 8)	*p* (Mann–Whitney U Test)
Age (mean month, range)	13.2 (1–108)	14.3 (0.4–166)	0.735
Sex (m:f)	10:9	4:4	0.938
Past history (−:+)	13:6	7:1	0.449
Whether or not the home of the place of discovery (−:+)	18:1	5:3	0.198
Whether found after bedtime (−:+)	18:1	1:7	<0.001
Estimated time from the last sighting to discovery (mean minutes, range)	254.8 (0–560)	17.3 (0–115)	<0.001
Bystander CPR (+:−)	7:12	3:5	0.979

CPR—cardiopulmonary resuscitation; ROSC—return of spontaneous circulation.

**Table 2:** Differences in CT findings of OHCA between non-ROSC and ROSC groups.

	Number of Positive Cases		
CT Findings	non-ROSC (*n* = 19)	ROSC (*n* = 8)	*p* (Fisher’s Exact Test)
Head			
Brain swelling	16	1	<0.001
Loss of cerebral gray-white matter differentiation	14	3	0.033
Hyperdense intracranial venous sinus	6	0	0.136
Lung			
Symmetrical consolidation/ground-glass opacity	18	4	0.017
Asymmetrical consolidation/ground-glass opacity	2	2	0.558
Mediastinum			
Cardiomegaly	16	2	0.006
Pericardial effusion	0	0	n/a
Hyperdense aortic wall	16	0	<0.001
Narrowed aorta	19	0	<0.001
Gas in the cardiac cavity, aorta, and superior vena cava	15	0	<0.001
Abdomen and pelvis			
Hepatomegaly	15	1	0.002
Dilated inferior vena cava	3	2	0.616
Dilated gastrointestinal tract	17	5	0.136
Gas in the upper abdominal organs	8	0	0.061
Soft tissue			
Subcutaneous fatty edema	0	0	n/a

CT—computed tomography; *n*—number; OHCA—out-of-hospital cardiac arrest; ROSC—return of spontaneous circulation.

## Data Availability

Raw data are available upon request.

## Supplementary Materials

The following supporting information can be downloaded at https://www.mdpi.com/article/10.3390/diseases12100261/s1, Table S1: Clinical information and CT findings of 19 pediatric OHCA patients with non-ROSC; Table S2: Clinical information and CT findings of 8 pediatric OHCA patients with ROSC.
