# Supplementary material for: Clinical Significance of Whole-Body Computed Tomography Scans in Pediatric Out-of-Hospital Cardiac Arrest Patients Without Prehospital Return of Spontaneous Circulation
Source: Diseases. 2024 Oct 20;12(10):261. doi: 10.3390/diseases12100261 (PMC11506920; doi:10.3390/diseases12100261)
Supplement: Supplementary file 1 [file diseases-12-00261-s001.zip › diseases-3217750-supplementary.pdf]

Table S1. Clinical information and CT findings of 19 pediatric OHCA patients with non-ROSC

Case no.	1	2	3	4	5	6	7	8	9	10	11	12	13	14	15	16	17	18	19	Detection rate (n)
Age/sex	2y/m	2y11m/f	2m/f	5m/m	9y/f	5y11m/f	1y2m/m	10m /f	2y7m/f	3m/f	5m/f	1y2m/m	2m/m	1m/m	1y4m/m	6y1m/m	4m/f	1m/m	9m/m	
Past history	none				asthma (currently untreated)	tubulinopathy	none		congenital cytomegalovirus infection, symptomatic epilepsy	none	neonatal asphyxia, transient tachypnea of the newborn	none			febrile convulsions, asthma	none			periventricular leukomalacia	
Scene of the find	sleeping on prone position at home			sleeping on right lateral position beside the bed	supine position with loss of consciousness at home	sleeping on prone position at home	prone position with the face wrapped in a blanket at home	sleeping on prone position at home							sleeping on supine position at home	loss of consciousness on the train	sleeping on supine position at home			

Estimated time from the last sighting to discovery	10m	7h30m	5h30m	4h	2m	5h40m	4h	7h30m	2h50m	1h20m	6h30m	3h	9h20m	4h25m	1h	0m	2h25m	8h	7h30m	
Bystander CPR	+	-	-	-	+	+	-	-	-	+	+	-	+	+	-	-	-	-	-	
ROSC at emergency department	-	-	-	-	-	-	-	-	-	-	-	-	-	-	-	-	-	-	-	
Timing of CT scanning	during CPR							after CPR	during CPR	after CPR	during CPR									
CT findings Head																				
Brain swelling	+	+	-	-	-	+	+	+	+	+	+	+	+	+	+	+	+	+	+	84
Loss of cerebra	-	+	+	+	-	+	-	-	+	+	+	+	+	-	+	+	+	+	+	74

l gray-white matter differentiation																				
Hyperdense intracranial venous sinus	-	+	-	+	-	-	-	-	-	-	+	-	-	-	+	-	-	+	+	32
Lung																				
Symmetrical consolidation/ground-glass opacity	+	+	+	+	+	+	-	+	+	+	+	+	+	+	+	+	+	+	+	95
Asymmetrical consolidation/ground glass opacity	-	-	-	-	-	+	+	-	-	-	-	-	-	-	-	-	-	-	-	11
Mediastinum																				

Cardiomegaly	-	+	+	+	+	-	-	+	+	+	+	+	+	+	+	+	+	+	+	84
Pericardial effusion	-	-	-	-	-	-	-	-	-	-	-	-	-	-	-	-	-	-	-	0
Hyperdense aortic wall	+	-	+	-	+	+	+	+	+	-	+	+	+	+	+	+	+	+	+	84
Narrowed aorta	+	+	+	+	+	+	+	+	+	+	+	+	+	+	+	+	+	+	+	100
Gas in the cardiac cavity, aorta, or superior vena cava	+	+	+	-	-	+	-	+	+	+	+	+	+	+	+	-	+	+	+	79
Abdomen and pelvis																				
Hepatomegaly	+	-	+	+	+	+	+	+	+	+	+	+	-	+	+	+	-	-	+	79

Dilated inferior vena cava	-	-	-	-	+	-	-	-	-	-	-	+	-	-	-	-	+	-	-	16
Dilated gastrointestinal tract	-	+	+	+	+	-	+	+	+	+	+	+	+	+	+	+	+	+	+	89
Gas in the liver, kidney, pancreas, or spleen	-	+	+	-	-	-	-	-	-	+	+	+	-	+	-	-	-	+	+	42
Soft tissue																				
Dorsal subcutaneous fatty edema	-	-	-	-	-	-	-	-	-	-	-	-	-	-	-	-	-	-	-	0

y - year
m - month
h - hour
CPR - Cardiopulmonary Resuscitation
CT - Computed Tomography
ROSC - Return of Spontaneous Circulation

Table S2. Clinical information and CT findings of 8 pediatric OHCA patients with ROSC

Case no.	1	2	3	4	5	6	7	8	Detection rate (n)
Age/sex	12m/m	10m/m	10m/m	12d/f	3y5m/f	4m/m	17y/f	13y10m/f	
Past history	none							hypertrophic cardiomyopathy	
Scene of the find	sleeping on prone position at home	choked while eating		sleeping on prone position at home	on the car to the hospital	choked while eating	fallen down while playing volleyball	lying down at school	
Estimated time from the last sighting to discovery	5m	0	0	1m	0	1h55m	0	10m	
Bystander CPR	+	-	-	-	+	-	-	+	
ROSC at emergency department	+	+	+	+	+	+	+	+	
Timing of CT scanning	during CPR								
CT findings									
Head									
Brain swelling	-	-	+	-	-	-	-	-	12.5

Loss of cerebral gray-white matter differentiation	+	-	+	-	-	+	-	-	37.5
Hyperdense intracranial venous sinus	-	-	-	-	-	-	-	-	0
Lung									
Symmetrical consolidation/ ground glass opacity	+	-	-	-	+	-	+	+	50
Asymmetrical consolidation/ ground-glass opacity	+	+	-	-	-	-	-	-	25
Mediastinum									
Cardiomegaly	-	-	-	-	-	-	+	+	25
Pericardial effusion	-	-	-	-	-	-	-	-	0
Hyperdense aortic wall	-	-	-	-	-	-	-	-	0
Narrowed aorta	-	-	-	-	-	-	-	-	0
Gas in the cardiac cavity, aorta, or superior vena cava	-	-	-	-	-	-	-	-	0
Abdomen and pelvis									

Hepatomegaly	-	+	-	-	-	-	-	-	12.5
Dilated inferior vena cava	-	-	-	+	-	-	-	+	25
Dilated gastrointestinal tract	+	-	+	+	+	+	-	-	62.5
Gas in the liver, kidney, pancreas, or spleen	-	-	-	-	-	-	-	-	0
Soft tissue									
Subcutaneous fatty edema	-	-	-	-	-	-	-	-	0

y - year
m - month
h - hour
CPR - Cardiopulmonary Resuscitation
CT - Computed Tomography
ROSC - Return of Spontaneous Circulation
